# Correlation Analysis of Sleep Quality and Youth Ischemic Stroke

**DOI:** 10.1155/2014/246841

**Published:** 2014-08-05

**Authors:** Shunqing Zhang, Cheng Chang, Juan Zhang, Bo Song, Hui Fang, YuMing Xu

**Affiliations:** ^1^Neurological Department of the First Affiliated Hospital of Zhengzhou University, Zhengzhou 450052, China; ^2^Neurological Department of Puyang People's Hospital, Puyang 457000, China; ^3^Human Anatomy Department of Zhengzhou University, Zhengzhou 450001, China; ^4^Nuclear Medicine Department of the Fifth Clinical Medicine Hospital of Beijing University, Beijing 100730, China

## Abstract

*Objective*. To study risk factors related to ischemic stroke (IS) in youth and the influence of sleep quality on youth ischemic stroke incidence. *Methods*. 223 patients aged 18 to 45 years who were admitted to Puyang People's Hospital from June 2011 to February 2013 with a first-ever ischemic stroke were selected as the research cases. 158 young people with a normal physical examination were selected as the control group. The Pittsburgh Sleep Quality Index (PSQI) questionnaire was used to analyse the correlation between sleep quality and youth IS incidence. The US National Institutes of Health Stroke Scale (NIHSS) and modified Rankin Scale (MRS) scores were used to assess cases' state of illness and prognosis three months after IS. *Results*. Univariate and multivariate logistic regression analysis showed that the association of these risk factors with youth IS incidence, from highest to lowest, was hypertension, hyperlipidaemia, smoking history, high homocysteine, the quality of sleep, family history of stroke, and alcoholism. Poor sleep quality ranked fifth among all risk factors and was positively correlated with poor prognosis for youth IS patients. *Conclusion*. The results of this study showed that sleep quality is an important factor in the pathogenesis and prognosis of youth IS.

## 1. Introduction

Ischemic stroke (IS) has become a high incidence, high morbidity, and high mortality disease in recent years, and its incidence is increasing significantly in young people [1]. Youth IS is defined as patients who are 18 to 45 years old with ischemic stroke, and it accounts for approximately 5 to 10% of all IS cases [2, 3]. Youth IS not only affects the patient's quality of life and work but also inflicts a heavy economic burden on families and society. Therefore, exploring IS risk factors and proactive prevention strategies are important. Youth IS risk factors and active prevention research on youth IS have attracted the attention of medical workers; however, risk factors reported in China are inconsistent with that in other countries [4–6]. Studies have shown that sleep quality has a close relationship with ischemic stroke incidence and prognosis [7]. Current research mainly focuses on the mechanisms of sleep disorders after cerebral infarction in youth IS patients. Few studies have explored the role of sleep quality in youth IS pathogenesis or investigated whether sleep quality is a cause of youth IS incidence and prognosis [8, 9]. This study extracted data on 223 youth IS patients from Puyang City People's Hospital to observe and analyse the relationship between sleep quality and youth IS, find new youth IS risk and prognostic factors, and provide clinical evidence for the prevention and treatment of youth IS.

## 2. Materials and Methods

### 2.1. Research Materials

#### 2.1.1. Selection of Study Subjects

In total, 223 patients aged 18 to 45 years who were admitted to Puyang People's Hospital from June 2011 to February 2013 with a first-ever ischemic stroke were selected as the research cases. Young people without obvious discomfort and symptoms (aged 18 to 45 years) were elected as the control group by normal physical examination in the same hospital. The study was approved by the Ethics Committee of Puyang City People's Hospital.

#### 2.1.2. Inclusion and Exclusion Criteria

Patient inclusion criteria were as follows: (1) fulfilment of 2007 AHS/ASA ischemic stroke clinical diagnostic criteria [10]; (2) hospitalization within 72 h after IS onset; (3) clear focal neurological deficit symptoms and signs lasting more than 24 h without relief; (4) head MRI diffusion sequences confirming a clear ischemic infarct; and (5) consciousness. Patient exclusion criteria were as follows: (1) cerebral haemorrhage by confirming head CT; (2) thrombolysis within the time window; (3) severe heart, liver, or kidney disease and/or cancer; (4) severe mental illness; (5) ischemic stroke history or sequela; (6) impairment in muscle strength and the ability to make informed life decisions, including suffering from orthopaedic, rheumatic, and/or mental disorders; and (7) failure to consent to follow-up or participation in the establishment of a database.

### 2.2. Research Methods

#### 2.2.1. Data Collection and Outcome Measures

Patients included in the observation group were hospitalized within 72 h after IS onset. Once hospitalized, details regarding patients' past medical, personal, and family histories were obtained and recorded. In addition, IS-related data (hypertension, hyperlipidaemia, smoking history, alcohol consumption history, sleep quality, high homocysteine, diabetes, heart disease, cerebrovascular disease, and family history) was collected from medical records. On the second day of hospitalization, patient improvement was monitored via vital signs, ECG, blood biochemistry tests, carotid ultrasound, the Pittsburgh Sleep Quality Index (PSQI) questionnaire score, the US National Institutes of Health Stroke Scale (NIHSS) score, and the modified Rankin Scale (MRS) score. Patients with mild sleep disorders were made nondrug spiritual comfort treatment. For that with severe sleep disorders were made short-term medication within hospitalization. For severe sleep disorder cases, PSQI scores were recorded as ≥7 (poor sleep quality). Admission time, treatment options, and treatment process for all cases were found to have no significant differences by Power-Test analysis. This ensured that the data collected from different cases were comparable.

A detailed explanation about youth IS pathogenesis and harm and common risk factors (hypertension, high cholesterol, smoking, alcoholism, sleep quality, high homocysteine, and diabetes) was provided to observation group members for health knowledge and education. Patients in the observation group were required to have the follow-up examination in the morning, with fasting, three months (90 ± 3 d) after discharge. Additionally, patients were encouraged by attending doctors to quit smoking, limit alcohol, control their diet, take medication regularly, monitor their blood pressure and biochemistry (lipids, glucose, homocysteine, and other targets), and record PSQI, NIHSS, and MRS scores during the period of follow-up examination (90 ± 3 d after discharge). A complete personal database file was established for all patients. The person responsible for data collection, data entry, and follow-up and management and quality control of the database conducted follow-up or home visits.

#### 2.2.2. Imaging Examination

All patients were admitted to the hospital after completion of cranial CT (computerized tomography), MRI (magnetic resonance imaging), neck vascular ultrasound, TCD (transcranial Doppler), MRA (magnetic resonance angiography), and other tests. Some patients also underwent a CTA (CT angiography) or DSA (digital subtraction angiography) examination.

### 2.3. Definitions of Risk Factors

1. Hypertension [11]: systolic blood pressure ≥ 140 mm Hg and/or diastolic blood pressure ≥ 90 mm Hg with a history of taking antihypertensive drugs.
2. Hyperlipidaemia [12]: total cholesterol (TCH) ≥ 5.70 mmol/L and/or triglyceride (TG) ≥ 2.04 mmol/L, high-density lipoprotein cholesterol (HDL-C) < 1.0 mmol/L, low-density lipoprotein cholesterol (LDL-C) > 3.2 mmol/L, lipoprotein(a) [LP(a)] > 300 mg/L, apolipoprotein Al (Apo-A1) > 1.09/L, and lipoprotein B100 (Apo-B100) > 1.09/L.
3. High homocysteine (Hcy) [13]: plasma Hcy levels of 6 to 15 *μ*mol/L (men < 60 years), 3 to 12 *μ*mol/L (women < 60 years), or 15 to 20 *μ*mol/L (men and women > 60 years).
4. Diabetes [14]: a history of diabetes, two fasting blood sugar readings of ≥ 7.0 mmol/L or two 2 h postprandial readings of ≥ 11.1 mmol/L after IS onset.
5. Smoking history: >10 cigarettes/d for >12 months or >20 cigarettes/d for >10 years even quitting <10 years.
6. Drinking history: >750 mL/d of beer, >10 mL/d of hard alcohol, or >360 mL/d of wine, in accordance with the World Health Organization (WHO) classification criteria.
7. Family history: history of cerebrovascular disease in the immediate family, such as parents, brothers, and sisters.
8. Sleep quality: quality of sleep over a one-month period as measured by the Pittsburgh Sleep Quality Index (PSQI). The PSQI is composed of 18 items that assess seven areas of sleep (sleep quality, time to fall asleep, sleep duration, sleep efficiency, sleep disorders, sleep medication, and daytime dysfunction). Each area is given a score of 0 to 3 points, yielding a cumulative PSQI score of 0 to 2l. Cumulative PSQI scores of < 7 points indicated good sleep, while PSQI scores of ≥ 7 indicated poor sleep [15].
9. Exercise: aerobic exercise at least twice per week for more than 30 min each time.

### 2.4. Prognosis and Outcome Assessment

Results of the modified Rankin Scale outcome assessment were divided into the following categories: MRS score < 3 (self-care), MRS score ≥ 3 (dependent on others), and MRS score = 6 (death) [16].

### 2.5. Statistical Analysis

SPSS10.0 was used for statistical analysis. Normally distributed variables were presented as the mean values (standard deviation), nonnormally distributed variables were presented as median values (*P*_25_, *P*_75_), and count data were presented as the number of cases (%). Univariate analysis was performed to examine descriptive statistics and to conduct *x* ^2^ tests for normally distributed continuous variables, *t*-tests, and nonparametric Mann-Whitney tests performed on the level of the variable. Compared variables of differences between observation and control group were screened by univariate analysis. Finally, a multivariate logistic regression was conducted within filtered compared variables (risk factors) to identify statistically significant variables associated with youth IS. *P* values of <0.05 were considered statistically significant.

## 3. Results

### 3.1. General Situation

The youth observation group consisted of 223 cases, 170 (76.2%) male and 53 (23.8%) female. The youth control group consisted of 158 cases, 111 (70.3%) male and 47 (29.7%) female.

### 3.2. Risk Factors Analysis for Observation Group and Youth Group

Univariate analysis showed that the following were risk factors for youth IS: hypertension, hyperlipidaemia, diabetes, family history of stroke, high homocysteine, quality of sleep, alcohol consumption, and smoking history. They all had statistical differences between observation and youth control group (*P* < 0.05). The proportion of cases with poor sleep quality in patients and in healthy young people was 47.1 and 29.1%, respectively, and the difference between the two groups was statistically significant (*P* < 0.001) (Table 1). However it is difficult to say how much the poor sleep quality was different between two groups. Seven risk factors (hypertension, hyperlipidaemia, family history of stroke, high homocysteine, quality of sleep, alcohol consumption, and smoking history) were screened by univariate analysis. They were made as independent variables and youth ischemic stroke was made as dependent variables. A multivariate logistic regression was conducted to identify statistically significant variables associated with youth IS. Arranged in order of significance, the five relative risk factors for youth IS, from highest to lowest, were hypertension, hyperlipidaemia, smoking, high homocysteine, and poor sleep quality (Table 2).

### 3.3. Short-Term Prognosis for Youth IS

Univariate analysis showed that the prognosis of young stroke patients included the following factors: NIHSS score, quality of sleep, and high homocysteine (*P* < 0.05) (Table 3). The aforementioned significant variables were put into logistic regression models. Poor sleep quality was associated with the three-month MRS scores of youth IS patients (OR: 1.829; 95% CI: 1.014–3.301). NIHSS score and high hyperlipidaemia were also associated with the three-month MRS scores of youth IS patients (Table 4).

## 4. Discussion

In recent years, the incidence of ischemic stroke in young people has increased and has received growing attention. The aetiology of IS is very complex; researchers have continued to investigate various risk factors for ischemic stroke with the aim of preventing youth IS by controlling its risk factors. Recently, the importance of identifying risk factors for ischemic stroke prevention has gained widespread concern. Youth IS risk factors include modifiable and nonmodifiable factors. Modifiable risk factors include smoking, drinking, hypertension, diabetes, hyperlipidaemia, high homocysteine, sleep quality, and eating habits. Nonmodifiable risk factors include age, gender, race, region of residence, and genetic factors. Studies conducted both in and out of China have shown that hypertension, hyperlipidaemia, and smoking are the most common risk factors for youth IS. Research shows that, out of China, the predominant risk factors were smoking and hypertension. In China, the predominant risk factors were high blood pressure and hyperlipidaemia [17].

Sleep is important to maintain the physiological processes of human life and for the protection of cortical inhibition. Adequate sleep is also essential to protecting brain cell energy metabolism. Studies have shown that sleep disorders are closely related to stroke risk factors such as diabetes, hypertension, and obesity. Research on the relationship between sleep quality and stroke has attracted widespread interest [18].

### 4.1. Relationship between Youth IS Onset and Sleep Quality

After comparing young patients and young healthy people by gender, age, condition, and rehabilitation of many risk factors, this study showed that male sex, hypertension, hyperlipidaemia, high blood homocysteine, diabetes, family history, poor quality of sleep, smoking, and alcoholism were associated with the incidence of stroke in young people. In this study, univariate analysis showed a statistically significant difference in sleep quality, with a higher proportion of poor sleep quality in youth IS patients (47.1%) compared with healthy individuals (29.1%). Results of the multivariate logistic regression analysis showed that poor sleep quality was the fifth strongest predictor of youth IS, indicating that this factor cannot be ignored and plays an important role in the pathogenesis of youth IS. The following reasons for poor sleep quality and insomnia were reported: pressure from work and life, poor self-control, addiction to the Internet, addiction to television, drinking, playing cards, excessive entertainment, modern nightlife, literacy, living alone, negative emotion, and mental health diagnosis. This study found that poor sleep quality is associated with the incidence of youth IS and is a powerful factor in youth IS onset. Attention to sleep quality provides guidance for youth IS prevention and has important clinical and social significance.

### 4.2. Relationship between Sleep Quality and Youth IS Short-Term Prognosis

For youth IS, neurological impairment and restoration of life skills are important prognostic indicators. There are many evaluation studies of stroke scales used to measure the severity of stroke, and, of these, the NIHSS has demonstrated the highest degree of validity and reliability and is the most widely used. In one study, the NIHSS score at three months was strongly associated with the degree of independence of patients [19]. In this study, the NIHSS score was an independent predictor of poor youth IS prognosis (OR: 2.846; 95% CI: 1.475–5.491). Epidemiological studies have shown that 15 to 35% of adults have sleep problems [20]. The level of sleep quality may differ across countries and regions due to various ethnic, geographical, economic, and cultural factors. Differences in the results of studies also have a strong relationship with the sampling method, survey group, and diagnostic criteria. This study investigated sleep quality as a prognostic variable and analysed its influence on youth IS prognosis. The results showed that poor sleep quality is associated with poor prognosis in youth IS patients (OR: 1.829; 95% CI: 1.014–3.301), suggesting that good quality sleep may improve the prognosis of youth IS patients.

This study showed that there was a greater proportion of young male IS patients compared with female patients. This finding is consistent with the existing Chinese literature. Both similarities and differences between Chinese and other areal research have been demonstrated in risk factors associated with youth IS shown in the areas of youth IS pathogenesis. Logistic multivariate analysis showed that sleep quality was the fifth strongest predictor of youth IS. Thus, poor sleep quality is a significant contributor to the pathogenesis of youth IS. Youth IS is influenced by many factors. Risk factors and results identified in Chinese and other areal research differ due to different regions, climates, foods, cultures, and living habits.

Youth IS prevention in patients with poor compliance, the resultant impact on families and society, and the increasing IS incidence are all worthy of concern. Deep discussion of the risk factors related to youth IS, prognostic risk factors, and preventive measures, in addition to actively improving youth IS patient compliance, is our imperative duty and mission as medical workers. Clinical work should promote youth IS patients to develop good habits, provide universal health education about stroke, and understand the risk factors, especially the dangers of poor sleep quality. Appropriate primary and secondary prevention can effectively reduce the incidence of stroke in young people.

The limitations of this study included presence of an insufficient sample size and lack of basic research on sleep quality and youth IS onset. From the perspective of evidence-based medicine, further scientific evaluation of the relationship between sleep quality and youth IS incidence and prognosis using larger study samples and a multicentre randomized experimental design is needed. Whether poor sleep quality is an independent causal risk factor of youth IS incidence and prognosis requires further discussion and study. The positive correlation found between the risk factors and incidence of youth IS must be confirmed by further studies.

## Figures and Tables

**Table 1:** Risk factors analysis for observation group and youth control group. By univariate analysis, smoking history, drinking history, sleep quality, hypertension, diabetes, hyperlipidaemia, family history of stroke, and high homocysteine were statistically different between youth IS patients and youth control cases (*P* < 0.05).

Risk factors	Observation group (*n* = 223)	Young control group (*n* = 158)	Statistic	*P*
Age (years)	38.26 ± 6.351	35.99 ± 6.660	1.856	0.174
Gender (male)	170 (76.2)	111 (70.3)	1.708	0.191
Exercise	21 (9.4)	23 (14.6)	2.392	0.122
Smoking history	97 (43.5)	36 (22.8)	17.461	0.000
Drinking history	111 (49.8)	52 (32.9)	10.744	0.001
Sleep quality	105 (47.1)	46 (29.1)	12.484	0.000
Hypertension	104 (46.6)	30 (19.0)	31.007	0.000
Diabetes	44 (19.7)	18 (11.4)	4.719	0.030
Hyperlipidaemia	74 (33.2)	33 (20.9)	6.925	0.009
Family history of stroke	79 (35.4)	33 (20.9)	9.420	0.002
High homocysteine	92 (41.3)	34 (21.5)	16.275	0.000

**Table 2:** Multivariate logistic regression analysis of risk factors for youth ischemic stroke. By multivariate logistic regression analysis, relative risk factors for youth IS, from highest to lowest, were hypertension, hyperlipidaemia, smoking history, high homocysteine, and poor sleep quality.

	OR	OR (95% CI)	*P*
Hypertension	3.153	1.874–5.304	0.000
Hyperlipidaemia	2.205	1.298–3.745	0.003
Smoking history	2.091	1.257–3.479	0.004
High homocysteine	2.051	1.230–3.421	0.006
Sleep quality	1.811	1.116–2.939	0.016
Family history of stroke	1.757	1.029–3.002	0.039
Drinking history	1.718	1.074–2.749	0.024

**Table 3:** Univariate analysis of short-term prognostic factors in youth IS patients. By univariate analysis, NIHSS score, poor sleep quality, and high homocysteine were statistically different between living self-care and living dependent patients (*P* < 0.05).

Factors	Daily living self-care (MRS < 3) (*n* = 150)	Living dependence (MRS ≥ 3) (*n* = 73)	Statistic	*P*
Gender (male)	115 (76.7)	55 (75.3)	0.048	0.827
Age	38.13 ± 6.25	38.55 ± 6.58	0.048	0.827
NIHSS	5.5 (3, 8)	9 (6, 15)	5.113	0.000
TC	1.72 ± 1.11	1.44 ± 0.93	3.580	0.06
TG	4.53 ± 1.38	4.45 ± 1.20	1.247	0.265
HDL	1.25 ± 0.49	1.28 ± 0.34	1.287	0.258
LDL	2.96 ± 1.04	3.04 ± 1.03	0.322	0.571
Admission fasting glucose	5.08 ± 2.54	5.20 ± 2.27	0.878	0.350
Sleep quality (poor)	62 (41.3)	43 (58.9)	6.085	0.014
High homocysteine	50 (34.5)	42 (57.5)	10.578	0.001

**Table 4:** Logistic regression analysis of prognostic factors in youth IS patients. By multivariate logistic regression analysis, association with the three-month MRS scores, from highest to lowest, were NIHSS score, high homocysteine and poor sleep quality.

Variable	OR	OR (95% CI)	*P*
NIHSS	2.846	1.475–5.491	0.002
Sleep quality (poor)	1.829	1.014–3.301	0.045
High homocysteine	2.232	1.226–4.062	0.009
